# Association of knowledge and beliefs with the misuse of antibiotics in parents: A study in Beirut (Lebanon)

**DOI:** 10.1371/journal.pone.0232464

**Published:** 2020-07-22

**Authors:** Narmeen Mallah, Danielle A. Badro, Adolfo Figueiras, Bahi Takkouche

**Affiliations:** 1 Department of Preventive Medicine, University of Santiago de Compostela, Santiago de Compostela, Spain; 2 Centro de Investigación Biomédica en Red de Epidemiología y Salud Pública (CIBER-ESP), Madrid, Spain; 3 Faculty of Health Sciences, American University of Science and Technology, Beirut, Lebanon; 4 Health Research Institute of Santiago de Compostela (IDIS), Santiago de Compostela, Spain; Public Library of Science, UNITED KINGDOM

## Abstract

**Background:**

Antibiotic resistance is a major public health concern. It has been associated with factors such as uncontrolled consumption, lack of knowledge, beliefs, and sociodemographic characteristics. Lebanon is characterized by high levels of antibiotic misuse, as almost half of the population self-medicates with antibiotics and over 30% of the antibiotics are dispensed without prescription. To-date, no studies determined adequately the association between knowledge, beliefs and antibiotic misuse in Lebanon.

**Objective:**

To assess the association between level of knowledge and beliefs about antibiotics, and antibiotic misuse in Lebanon.

**Methods:**

We conducted a cross-sectional study among 1,421 parents of schoolchildren using an anonymous self-administered Knowledge, Attitude and Practices questionnaire. The participants´ level of agreement with each item of knowledge and beliefs was measured using a Likert-type (0–10) scale. Misuse practices in the last month were detected through a series of questions aimed at determining the level of compliance with physicians´ instructions in terms of dosage and duration. Data were analyzed using logistic regression.

**Results:**

277 participants (16%) acknowledged using antibiotics in the previous month, 41% of whom showed at least one misuse behavior. Misconceptions and beliefs about antibiotics substantially increased the odds of their misuse. For instance, participants who believed that antibiotics were effective in treating viruses, cold, or sore throat infections were twice as likely to misuse antibiotics [Adjusted Interquartile Odds Ratio (aIqOR): 2.08 (95%CI: 1.32, 3.19), aIqOR: 1.81 (95%CI: 1.41, 2.29), aIqOR: 2.19 (95%CI: 1.61, 2.93), respectively]. Parents who usually keep antibiotics at home antibiotics for future use were more likely to misuse antibiotics [aIqOR: 2.44 (95%CI: 1.68, 3.46)].

**Conclusions:**

Our findings indicate that the low level of knowledge and the existence of erroneous beliefs about antibiotics are associated with increased odds of antibiotic misuse. Key elements including rationale prescription and control of dispensing should be addressed when designing educational campaigns against antibiotic misuse.

## Introduction

Antibiotic resistance is a growing global public health concern, with heavy economic and social burdens. Antibiotic-resistant bacteria cause 700,000 deaths per year worldwide, and it is predicted that resistance will be more lethal than cancer by 2050. [1] The misuse of antibiotics through excessive medical prescriptions and self-medication is an extremely important cause of antibiotic resistance. Indeed, the global consumption of antibiotics increased by 65% between 2000 and 2015, and this steadily increasing trend is mainly observed in low- and middle-income countries. [2] The majority of these antibiotics are consumed outside hospitals, and self-medication with antibiotics is common practice worldwide, except in northern Europe and North America where it is strictly regulated. [3] To prevent further expansion of antibiotic resistance, regional and global action plans were initiated and were mainly based on improving awareness tools that took into consideration critical criteria such as demographic characteristics of consumers, as well as their level of knowledge and beliefs with regard to antibiotic misuse. [4–8]

Population mobility is another important factor contributing to the globalization of the antibiotic resistance threat, as international travelers disseminate resistant bacteria across geographical boundaries. [3, 9–10] In that regard, Lebanon is a country with high immigration rate which has been receiving millions of refugees, mainly from neighboring Arab countries where antibiotic misuse is also very frequent. [11] Antibiotic use and dispensing without medical prescription are frequent in Lebanon. [12–15] Consequently, it is of utmost importance to implement measures that would improve the rational use of antibiotics in the country. Therefore, in the current study, we aimed at determining, through measures of association, the relation between the level of knowledge and beliefs about antibiotics and different aspects of antibiotic misuse in Lebanon. This could prove useful in designing educational campaigns oriented to enhance the awareness towards antibiotics and their rationale use.

## Materials and methods

### Study setting

A complete list of all private and public schools in the Beirut area was compiled and an invitation to participate in our study was sent to each school’s administration. 11 schools accepted our invitation and the parents of schoolchildren attending those schools were enrolled in this study and were provided with a printed copy of the questionnaire.

Our study was authorized by the Lebanese Ministry of Education and Higher Education and approved by the Institutional Review Board at the American University of Science and Technology (AUST-IRB-20180202-01).

### Sample size calculation

We calculated the sample size based on the following assumptions: alpha = 0.05, power: 0.8, ratio exposed/non exposed: 2, Odds Ratio to be detected: 2. With these assumptions, the needed number of respondents was 1052 subjects.

### Data collection

Schools were contacted by telephone or electronic mail, or were visited, in order to explain the study purpose and obtain the approval of schools’ directors to circulate the questionnaire among the community of parents. Before delivering it to parents, the questionnaire was reviewed by the board at every school, and parents were informed about the study objectives and the expected delivery date of the questionnaire by the director’s office.

As the questionnaire was anonymous, a written informed consent was neither necessary nor reasonable as it would have defeated the objective of anonymity. We therefore placed the following statement in the first page of the questionnaire (S1 File): “By sending this questionnaire back to us with your answers, you are giving your consent to participate in this study.” In the introduction of the questionnaire, we also stated: “You will NOT be asked about your name, address or any other information that reveals your identity. In addition, the answers received from all participants will be pooled into an anonymized database, where all given information will be kept confidential and will be eliminated at the end of the study.”

The questionnaire was then delivered to the parents in the main two languages of instruction used at the school (Arabic and English, or Arabic and French). A questionnaire was delivered to every household in a sealed envelope given to only one child of the family attending the school. Only one of the parents (either the mother or the father) was requested to answer the questionnaire. The parents were also notified about the questionnaire delivery in the same day in order to assure that they received the questionnaire. An extensive follow up was conducted to maximize the response rate. Questionnaires were collected five days after they were provided to parents.

Data were collected at different time points: November 2018 and January 2019. The corresponding dataset is available in the data repository FigShare https://doi.org/10.6084/m9.figshare.9929897.v1

### Measures

We selected relevant items from published questionnaires that preferably underwent a validation process previously. [5, 16–19] In addition, to identify the most frequent misuse practices, as well as the knowledge and beliefs factors that might be related to these practices, three focus group sessions were held with a total of 18 parents of schoolchildren of different demographic characteristics. Health professionals were excluded from the focus groups. The items were then selected, tailored whenever required for better comprehension and/or better suitability for the Lebanese population, and new questions were developed and added. Subsequently, experts in pharmacology, psychology, epidemiology and professionals with experience in the design of Knowledge, Attitude and Practice (KAP) questionnaires reviewed the face and content validity of the questionnaire in order to ensure that the items designed tackled all aspects of antibiotic misuse in Lebanon. Syntax of questions was altered accordingly.

Using antibiotics without medical prescription, shortening the duration of treatment with antibiotics, saving or sharing leftover antibiotics, skipping a dose of antibiotics or changing the prescribed dose were considered as practices of antibiotic misuse.

The level of agreement with the knowledge and beliefs statements was measured using a scale of zero (total disagreement) to 10 (total agreement). For the items assessing attitude to antibiotics and demographic characteristics, respondents were given a set of possible answers to choose from. The questionnaire was initially written in English then translated forward and backward by bilingual researchers into Arabic and French languages. The questionnaire was designed using Remark Office OMR software (Remark Office OMR 2014, version 9.2.0.20) to allow for automated optical reading of the data, thereby minimizing errors during data entry. The English version of the questionnaire is available as an online supplement (S1 File). Subsequently, a pilot testing of the questionnaires was conducted on 20 parents to identify any comprehension-related issues and adjust the syntax accordingly.

### Statistical analysis

To assess the reproducibility of the instrument, the questionnaire was administered to 60 subjects on two occasions, with a four-week interval. The intraclass correlation coefficients (ICCs) with their 95% CI and the weighted kappa indexes were calculated for variables with 0–10 scale outcomes and for nominal variables, respectively. Items showing an ICC value or weighted kappa index ≥ 0.4 were considered acceptable and retained in the final questionnaire.

In this study, six different outcomes were used to assess the misuse of antibiotics. The first outcome assessed antibiotic use without medical prescription and was determined by asking the following question “Q20. Who prescribed or recommended the use of antibiotics to you?”. The second outcome addressed the shortening of antibiotic therapy and was assessed through the question “Q21. The last time you had to take antibiotics; did you complete the course of treatment?”. The third outcome assessed whether unused antibiotics were saved or shared, using the question “Q22. What did you do with the antibiotics that were left unused?”. The fourth outcome assessed whether in case of a missed dose, the subsequent one was doubled or taken when remembered. The question used was “Q24. What did you do when you skipped a dose of your antibiotics?”. The fifth outcome assessed whether the parent modified the dose without medical advice and whether it was an increase or a decrease in the dose. We used two questions: “Q25. The last time you had to take antibiotics, did you change the dose on your own (without medical advice)” and “Q26. What did you do when you changed your dose of antibiotics on your own (without medical advice)?”. In the sixth outcome, the occurrence of any of the previously mentioned five misuse behaviors was evaluated.

The independent variables consisted of each item of knowledge, attitude or behavior included in the questionnaire. Covariates included age, sex, financial status, educational level, area of residence, alcohol consumption, access to medical care facilities, and frequency of telephone medical consultation.

Associations between independent variables and misuse of antibiotics were modeled using multiple logistic regression. Potential confounders were introduced consecutively into the model, and those that modified the value of the adjusted odds ratio by at least 10% were retained in the final model. Only individuals with complete data on the variables included in the model were analyzed. Finally, the adjusted interquartile odds ratio (aIqOR) were calculated to measure the effect of the exposure change from the 25th to the 75th percentile.

## Results

Out of 4,460 questionnaires distributed, a total of 1,460 (32.7%) were answered by the parents. 39 questionnaires were excluded as they were incompletely answered. We observed that these 39 excluded subjects had very low educational level (unschooled or primary education). The demographic characteristics of the parents included in the analysis are indicated in Table 1.

**Table 1:** Participants´ demographic characteristics.

	Characteristic	Total (N = 1421)	Any Misuse in the last month (N = 94)
**Gender**			
	Male	276 (19.4%)	22 (23.4%)
	Female	1092 (76.8%)	69 (73.4%)
	Missing	53 (3.7%)	3 (3.2%)
**Age**			
	<38 years	355 (25.0%)	37 (39.4%)
	39–42 years	227 (16.0%)	14 (14.9%)
	43–47 years	313 (22.0%)	13 (13.8%)
	> = 48 years	259 (18.2%)	14 (14.9%)
	Missing	267 (18.8%)	16 (17.0%)
**Marital status**			
	Married	1327 (93.4%)	87 (92.6%)
	Other	74 (5.2%)	6 (6.4%)
	Missing	20 (1.4%)	1 (1.1%)
**Educational level**			
	Until high school	252 (17.7%)	31 (33.0%)
	University	1157 (81.4%)	62 (66.0%)
	Missing	12 (0.8%)	1 (1.0%)
**Spouse educational level**			
	Until high school	313 (22.0%)	36 (38.3%)
	University	1028 (72.3%)	53 (56.4%)
	Missing	80 (5.6%)	5 (5.3%)
**Number of family members**			
	2–4	555 (39.1%)	36 (38.3%)
	5–6	715 (50.3%)	50 (53.2%)
	>6	129 (9.1%)	6 (6.4%)
	Missing	22 (1.5%)	2 (2.1%)
**Family income**			
	<500$	21 (1.5%)	2 (2.1%)
	500$ - 1499$	260 (18.3%)	34 (36.2%)
	1500–2500$	223 (15.7%)	17 (18.1%)
	>2500$	808 (56.9%)	36 (38.3%)
	Missing	109 (7.7%)	5 (5.3%)
**Consulting a doctor**			
	Rarely or never	155 (10.9%)	8 (8.5%)
	Sometimes	756 (53.2%)	63 (67.0%)
	Always	493 (34.7%)	22 (23.4%)
	Missing	17 (1.2%)	1 (1.1%)
**Reasons for not always consulting a doctor**			
	No need	390 (27.4%)	24 (25.5%)
	Fear	29 (2.0%)	2 (2.1%)
	No money	127 (8.9%)	20 (21.3%)
	No time	321 (22.6%)	26 (27.7%)
	Long waiting time	166 (11.7%)	15 (16%)
	No near clinic	53 (3.7%)	4 (4.3%)
**Ever received medical consultation over the phone**			
	No	491 (34.6%)	30 (31.9%)
	Yes	892 (62.8%)	62 (66%)
	Missing	38 (2.7%)	2 (2.1%)

In our study population, 64.1% of the participants did not always consult the doctor when they were sick; 8.9% of them due to financial reasons and 34.3% because of a lack of time. More than 60% of the respondents tended to consult the doctor over the phone and 27% believed that there was no need for always visiting the doctor.

### Patterns of antibiotic misuse

In the month preceding the study, 277 (16%) parents had used antibiotics and 94 (41.4%) of them had shown at least one misuse behavior. The most frequent misuse behaviors consisted in using antibiotics without medical prescription (22.5%), keeping antibiotic leftovers for future use or sharing them with other individuals (22%), and doubling the dose of antibiotics or taking it when remembered, in case a dose was skipped (10.6%). The associations between the items of knowledge and beliefs with each pattern of misuse of antibiotics were calculated after adjusting for gender and age. These variables were kept in the final model due to their biologic relevance and their known potential for confounding in epidemiologic studies. These associations are presented below.

### Misconceptions and misbeliefs about the function of antibiotics is associated with their misuse

Parents who thought that antibiotics were effective against viruses were twice as likely to present any of the antibiotic misuse behaviors [aIqOR: 2.08 (95% CI: 1.32, 3.19)] (Table 2). This misconception was strongly associated with using antibiotics without medical prescription [aIqOR: 2.83 (95% CI: 1.61, 5.33)], shortening the course of treatment [aIqOR: 2.66 (95% CI: 1.07, 6.27)], and modifying the dose without medical advice [aIqOR: 5.94 (95% CI: 1.23, 28.04)] (Table 3).

**Table 2:** Association of knowledge and attitudes with any misuse of antibiotics in 1392 parents of schoolchildren.

Knowledge or attitude statements	Percentile of responses			No misuse (N)	Any Misuse (N)	alqOR* (95%CI)
	25%	50%	75%			
Antibiotics are effective against viruses	0	4	7	1171	86	2.08 (1.32, 3.19)
Each type of infection needs a different antibiotic	8	10	10	1174	87	1.17 (0.94, 1.44)
Antibiotics can kill the bacteria that normally live on the skin and in the gut	5	8	10	1138	85	1.16 (0.77, 1.61)
If antibiotics are consumed in excess, they won’t work when they are really needed	8	10	10	1168	85	0.96 (0.81, 1.14)
When I get a cold, I take antibiotics to help me feel better faster	0	0	4	1187	88	1.81 (1.41, 2.29)
I expect my doctor to prescribe antibiotics if I suffer from common cold or flu symptoms	0	5	9	1180	88	1.42 (0.91, 2.36)
When I have a sore throat, I prefer to use an antibiotic	0	0	5	1187	88	2.19 (1.61, 2.93)
If I feel side effects during a course of treatment of antibiotics, I should stop taking them as soon as possible	5	9	10	1170	86	1.40 (0.95, 2.01)
It is good to be able to get antibiotics from relatives or friends without having to see a medical doctor	0	0	0	1186	88	1.00 (1.00, 1.00)
I take the antibiotics according to the doctor’s instructions	10	10	10	1186	86	1.00 (1.00, 1.00)
I prefer to keep antibiotics at home in case there is a need for them later	0	2	6	1181	86	2.44 (1.68, 3.46)
I trust the doctor’s decision if s/he decides to prescribe or not prescribe antibiotics	8	9	10	1183	87	0.88 (0.76, 1.04)
If I believe that I need an antibiotic and the doctor did not prescribe it, I will get it at the pharmacy without a prescription	0	0	2	1180	87	1.51 (1.35, 1.72)
Doctors often explain clearly to the patient the reasons for prescribing or not prescribing antibiotics	5	7	9	1180	86	0.75 (0.57, 1.00)
Doctors often explain clearly to the patient the instructions for the use of antibiotics	5	8	10	1179	86	0.66 (0.47, 0.90)
When you buy antibiotics, the pharmacist tells you about the importance of correct therapeutic compliance/adherence	3	6	9	1181	87	1.27 (0.83, 1.87)

(*): adjusted for gender and age

**Table 3:** Association of knowledge and attitudes with the different aspects of antibiotic´s misuse in parents of schoolchildren.

Knowledge or attitude statements	Unprescribed use (N = 1,361)		Shortened treatment (N = 1,376)		Changed dose (N = 1,293)		Improper action when skipping a dose (N = 1,295)		Storing or sharing leftovers (N = 1,298)	
	Events №	alqOR* (95%CI)	Events №	alqOR* (95%CI)	Events №	alqOR* (95%CI)	Events №	alqOR* (95%CI)	Events №	alqOR* (95%CI)
Antibiotics are effective against viruses	47	2.83 (1.61, 5.33)	23	2.66 (1.07, 6.27)	10	5.94 (1.23, 28.04)	24	1.71 (0.75, 3.58)	46	1.71 (1.00, 3.19)
Each type of infection needs a different antibiotic	49	1.17 (0.86, 1.59)	23	1.28 (0.77, 2.07)	11	0.77 (0.52, 1.17)	24	1.14 (0.76, 1.77)	46	1.06 (0.81, 1.42)
Antibiotics can kill the bacteria that normally live on the skin and in the gut	49	0.86 (0.53, 1.40)	22	0.86 (0.73, 1.76)	11	0.70 (0.25, 1.84)	24	1.16 (0.56, 2.49)	45	1.40 (0.82, 2.49)
If antibiotics are consumed in excess, they won’t work when they are really needed	45	0.94 (0.76, 1.17)	21	0.86 (0.64, 1.17)	9	0.72 (0.50, 1.04)	22	1.08 (0.72, 1.59)	43	0.98 (0.76, 1.21)
When I get a cold, I take antibiotics to help me feel better faster	49	2.29 (1.69, 3.22)	23	2.36 (1.52, 3.73)	11	4.42 (2.14, 9.38)	24	1.94 (1.26, 3.13)	46	2.22 (1.57, 3.04)
I expect my doctor to prescribe antibiotics if I suffer from common cold or flu symptoms	49	1.42 (0.76, 2.77)	23	3.52 (1.30, 10.60)	11	1.20 (0.29, 4.79)	24	1.20 (0.52, 3.00)	47	1.42 (0.76, 2.77)
When I have a sore throat, I prefer to use an antibiotic	49	3.30 (2.29, 5.00)	23	2.19 (1.22, 3.86)	11	4.01 (1.69, 9.54)	24	1.34 (0.73, 2.39)	47	2.10 (1.40, 3.05)
If I feel side effects during a course of treatment of antibiotics, I should stop taking them as soon as possible	47	2.49 (1.28, 4.83)	23	2.70 (1.00, 7.59)	10	1.28 (0.42, 3.86)	24	1.05 (0.56, 2.10)	46	1.16 (0.70, 1.84)
It is good to be able to get antibiotics from relatives or friends without having to see a medical doctor	49	1.00 (1.00, 1.00)	23	1.00 (1.00, 1.00)	11	1.00 (1.00, 1.00)	24	1.00 (1.00, 1.00)	47	1.00 (1.00, 1.00)
I take the antibiotics according to the doctor’s instructions	47	1.00 (1.00, 1.00)	23	1.00 (1.00, 1.00)	10	1.00 (1.00, 1.00)	24	1.00 (1.00, 1.00)	50	1.00 (1.00, 1.00)
I prefer to keep antibiotics at home in case there is a need for them later	47	3.64 (2.19, 6.05)	23	2.08 (1.06, 4.00)	10	5.05 (1.59, 16.78)	24	1.06 (0.53, 2.08)	46	3.30 (1.97, 5.29)
I trust the doctor’s decision if s/he decides to prescribe or not prescribe antibiotics	48	0.86 (0.71, 1.08)	23	0.83 (0.61, 1.12)	11	0.59 (0.41, 0.85)	24	0.89 (0.71, 1.37)	47	0.88 (0.71, 1.10)
If I believe that I need an antibiotic and the doctor did not prescribe it, I will get it at the pharmacy without a prescription	48	1.64 (1.39, 1.90)	23	1.49 (1.19, 1.88)	11	1.64 (1.21, 2.25)	24	1.51 1.21, 1.88)	47	1.54 (1.32, 1.82)
Doctors often explain clearly to the patient the reasons for prescribing or not prescribing antibiotics	47	0.63 (0.43, 0.92)	23	0.66 (0.39, 1.13)	11	0.60 (0.27, 1.26)	23	1.22 (0.69, 2.22)	46	0.89 (0.60, 1.36)
Doctors often explain clearly to the patient the instructions for the use of antibiotics	47	0.59 (0.37, 0.90)	23	0.59 (0.31, 1.16)	11	0.44 (0.17, 1.10)	23	0.77 (0.39, 1.54)	46	0.62 (0.39, 1.00)
When you buy antibiotics, the pharmacist tells you about the importance of correct therapeutic compliance/adherence	48	2.44 (1.27, 4.61)	23	1.50 (0.65, 3.46)	11	3.81 (0.89, 16.16)	24	1.27 (0.57, 2.84)	47	1.42 (0.78, 2.57)

(*): adjusted for gender and age

Parents who thought antibiotics treat colds were at substantially higher odds of any misuse behavior [aIqOR: 1.81 (95% CI: 1.41, 2.29)] (Table 2). Specifically, they were more prone to use antibiotics without prescription [aIqOR: 2.29 (95% CI: 1.69, 3.22)], stop taking antibiotics as soon as they felt better [aIqOR: 2.36 (95% CI: 1.52, 3.73)], double the subsequent dose of antibiotics or postpone their intake when forgotten [aIqOR: 1.94 (95% CI: 1.26, 3.13)], store unused antibiotics or share them with other individuals [aIqOR: 2.22 (95% CI: 1.57, 3.04)], and modify doses without medical advice [aIqOR: 4.42 (95% CI: 2.14, 9.38)] (Table 3).

The odds of misusing antibiotics were more than twice higher among parents who usually take antibiotics when they suffer from a sore throat [aIqOR: 2.19 (95% CI: 1.61, 2.93)] (Table 2). These parents were three times as likely to self-prescribe antibiotics [aIqOR: 3.30 (95% CI: 2.29, 5.00)]. Moreover, they were more susceptible to shortening the course of treatment [aIqOR: 2.19 (95% CI: 1.22, 3.86)], changing the prescribed dose of antibiotics [aIqOR: 4.01 (95% CI: 1.69, 9.54)], and saving the unused antibiotics or sharing them with someone [aIqOR: 2.10 (95% CI: 1.40, 3.05)] (Table 3).

### Storage of leftover antibiotics and self-prescription are related to antibiotic misuse

Participants who usually keep antibiotics at home for future use were more than twice as likely to misuse antibiotics [aIqOR: 2.44 (95% CI: 1.68, 3.46)] (Table 2). They were even at higher odds of using antibiotics without medical prescription [aIqOR: 3.64 (95% CI: 2.19, 6.05)], and modifying the dose of antibiotics without any prior medical advice [aIqOR: 5.05 (95% CI: 1.59, 16.78)] (Table 3).

Similar results were obtained for subjects who tends to buy antibiotics without medical prescription when they believe that they need a drug-based treatment that the physician did not prescribe (Tables 2 and 3).

### Patient-physician adequate communication is associated with a lower frequency of antibiotic misuse

Parents who believed that the physician explained clearly the motives for prescribing or not prescribing antibiotics were less likely to use antibiotics without medical prescription [aIqOR: 0.63 (95% CI: 0.43, 0.92)] (Table 3). The pattern was similar when parents believed that the physician explained clearly the instructions of use of antibiotics. These parents were less likely to misuse antibiotics in general [aIqOR: 0.66 (95% CI: 0.47, 0.90)] and to self-medicate with antibiotics [aIqOR: 0.59 (95% CI: 0.37, 0.90)] (Tables 2 and 3).

## Discussion

This study is the first to quantitatively assess the relation between the level of knowledge and beliefs, and the misuse of antibiotics in Lebanon, using measures of association. We showed that the misuse of antibiotics in Lebanon is strongly associated with the level of knowledge and the beliefs of the population. Furthermore, preference for storing antibiotic leftovers was identified as an attitude leading to antibiotic misuse. In contrast, trusting the physician´s decision, believing that the physician explained clearly the motives for prescribing or not prescribing antibiotics, and believing that the pharmacist explained clearly the instructions of use of antibiotics and their correct therapeutic compliance, were associated with a lower frequency of misuse.

Our findings have important implications. They confirm that the prevalence of antibiotic misuse remains high in the country, since 41% of the participants who took antibiotics in the month prior to the survey showed at least one misuse practice. Previous studies had shown that 22% to 51% of the Lebanese self-medicated themselves with antibiotics. [12, 13] Furthermore, our results indicate that self-prescription and saving antibiotic leftovers were the most frequent misuse behaviors among the Lebanese community. Our results also suggest that there was over-prescription by physicians, poor therapeutic compliance, and ease of access to antibiotics in Lebanon. Previous studies showed that, in Lebanon, more than half of the antibiotics were inadequately prescribed in terms of motives for prescription, appropriateness of the type of antibiotics, dosage and duration [14], and that more than one-third of these drugs were dispensed without medical prescription. [15, 20]

To date, the determinants of inappropriate antibiotic prescription have not been evaluated in Lebanon specifically. However, inappropriate antibiotic prescription practices have been widely reported, including in developed countries. [21, 22] In general, the main reason for improper antibiotic prescription seems to be the excessive complacency with the patients’ demands. [17] On the other side, pharmacists in Lebanon claim that the main motive for dispensing antibiotics without medical prescription is the patients´ inability to afford a visit to the doctor. This could be a consequence of the lack of social security coverage for around 52% of the Lebanese people. [23] We also showed that more than half of the study population does not always visit the doctor in case of sickness. These figures are alarming, especially with the increasing number of pharmacies and the continuous trust of the Lebanese parents in receiving antibiotics prescribed by a pharmacist. [24] A considerable concern is the pharmacists’ belief that some antibiotics are harmless, and their will to retain customers by delivering antibiotics without any medical prescription to increase their economic benefit. [20] In our study, parents showed considerable misunderstanding of the role of antibiotics. In fact, half of the parents thought that antibiotics cured viral infections and 75% expected to receive an antibiotic prescription when they suffered from common cold and preferred to keep antibiotics at home in case of future need. These misconceptions could have favored the misuse of antibiotics. Our findings are in line with other regional and international studies that reported an association between misconceptions about antibiotics and their misuse. [25–28]

The present study has its strengths and limitations. To our knowledge, it is the largest study that was ever conducted in the country on this topic though we cannot disregard the fact that only one-third of the parents answered the questionnaire, which may have increased the possibility of response bias. However, as observed in some studies, non-respondents are more likely to have poorer socio-economical levels and health profiles than the respondents. [29, 30] Therefore, they are more susceptible to misuse antibiotics. Our results would then have been more extreme with a higher response rate.

Previous studies in similar settings in Lebanon have mainly relied on convenience sampling. [12, 13] Our study population cannot be considered as a representative sample of the population of Beirut, however this should not alter our results since measures of association (OR) are independent of representativeness. [31] In addition, restricting the assessment to antibiotic use in the month that preceded the administration of the questionnaire, represented a short recall period and thus decreased the risk of recall bias.

Another potential limitation of our study is its cross-sectional design. Theoretically, this design does not allow inferring causal effects as the outcome (misuse of antibiotics) may have preceded the exposure (knowledge on antibiotics). However, in KAP studies, this is unlikely to occur as knowledge and beliefs, in general, are formed long before the misuse practices. Finally, we cannot ignore the possibility of overestimating the statistical significance due to multiple comparisons.

Since patients´ demands and perceptions strongly contribute to inappropriate prescription and use, as well as to antibiotic dispensing without medical prescription, we believe that interventions aiming at promoting proper use of antibiotics should include public awareness, implementation of regulations regarding the sale of antibiotics without medical prescription, the dispensing of exact quantities as indicated in the medical prescription, as well as enforcing laws for antibiotic prescription according to international clinical guidelines. In this regard, it has been shown that modern communication methods such as social media platforms and web-based initiatives proved useful in improving the knowledge of the population on antibiotic medication. [32]

## Conclusion

In the present study we identified the factors (knowledge and beliefs of the population) that are associated with the behavior towards antibiotics. Our results could prove useful for countries that share a similar situation of antibiotics´ improper use. We showed that there is a need for educational campaigns that improve the awareness of the population towards the proper use of antibiotics and the consequences of their misuse. Future studies assessing country-specific determinants of antibiotic misuse, such as country wealth and healthcare system factors would be useful for the implementation of comprehensive multilevel interventional programs aimed at limiting the spread of antibiotic resistance.

## Supporting information

S1 File Questionnaire applied to collect data about the knowledge, attitudes and practices towards antibiotics in Lebanon. (PDF)
