# Supplementary material for: Association of knowledge and beliefs with the misuse of antibiotics in parents: A study in Beirut (Lebanon)
Source: PLoS One. 2020 Jul 22;15(7):e0232464. doi: 10.1371/journal.pone.0232464 (PMC7375529; doi:10.1371/journal.pone.0232464)
Supplement: S1 File — (PDF) [file pone.0232464.s001.pdf]

Mom, Dad, Participate in this Survey and Help Make Our Society Healthier!

Dear parents,
The University of Santiago de Compostela in Spain and the American University of Science and Technology in Lebanon, invite you to participate in the first study about determining the factors leading to the misuse of antibiotics.

Why should you participate?
You, the parents, represent a large and important fraction of the Lebanese population. Therefore, we highly encourage you to participate in our study in order to help the relevant authorities establish health strategies that would diminish the problem of intentional and unintentional medicines' misuse. That would **reflect positively on your health and on your children's**. All that is needed from you is a little bit of your time!

How can you participate?
To participate in this study, you are kindly asked to answer this questionnaire and return it back to the school. **You may respond to the questionnaire regardless if you are using the medicines correctly or not**. **You will NOT be asked about your name, address or any other information that reveals your identity.** In addition, the answers received from all participants will be pooled into an **anonymized** database, where all given information will be kept **confidential** and will be eliminated at the end of the study.

Only one of the parents (the mother or the father) should answer the questionnaire. Participation in the study is **voluntary, and doesn't entail any financial costs**.

By sending this questionnaire back to us with your answers, you are giving your consent to participate in this study.

Thank you for your participation!

Code of the questionnaire: ____________________ Date: ________________

To choose an answer, please fill in <u>COMPLETELY</u> the corresponding circle(s): CORRECTLY answered: ● INCORRECTLY answered: ⊠ ∅

Please answer on the following questions about ***ANTIBIOTICS*** (Amoxicillin, Cefixime, Ciprofloxacin, Levofloxacin, Penicillin, etc.)

On a scale of 0 to 10 points, choose a number representing your level of agreement on the statements below. "0" (zero) means you totally disagree while "10" means that you agree completely.
Please evaluate the below statements **<u>REGARDLESS</u>** of whether you are using *antibiotics* **<u>OR NOT</u>**

Example: Practicing sports benefits health ⓪ ① ② ③ ④ ⑤ ⑥ ⑦ ● ⑨ ⑩

	Disagree ← → Agree
**1.** ***Antibiotics* are effective against viruses**	⓪ ① ② ③ ④ ⑤ ⑥ ⑦ ⑧ ⑨ ⑩
**2. When I get a cold, I take *antibiotics* to help me feel better faster**	⓪ ① ② ③ ④ ⑤ ⑥ ⑦ ⑧ ⑨ ⑩
**3. If I feel better after a few days, I sometimes stop taking my *antibiotics* before completing the course of treatment**	⓪ ① ② ③ ④ ⑤ ⑥ ⑦ ⑧ ⑨ ⑩
**4. I expect my doctor to prescribe *antibiotics* if I suffer from common cold or flu symptoms**	⓪ ① ② ③ ④ ⑤ ⑥ ⑦ ⑧ ⑨ ⑩
**5. It is good to be able to get *antibiotics* from relatives or friends without having to see a medical doctor**	⓪ ① ② ③ ④ ⑤ ⑥ ⑦ ⑧ ⑨ ⑩
**6. When I have a sore throat I prefer to use an *antibiotic***	⓪ ① ② ③ ④ ⑤ ⑥ ⑦ ⑧ ⑨ ⑩
**7. Each type of infection needs a different *antibiotic***	⓪ ① ② ③ ④ ⑤ ⑥ ⑦ ⑧ ⑨ ⑩
**8.** ***Antibiotics* can kill the bacteria that normally live on the skin and in the gut**	⓪ ① ② ③ ④ ⑤ ⑥ ⑦ ⑧ ⑨ ⑩
**9. If I feel side effects during a course of treatment of *antibiotics*, I should stop taking them as soon as possible**	⓪ ① ② ③ ④ ⑤ ⑥ ⑦ ⑧ ⑨ ⑩
**10. I take the *antibiotics* according to the doctor's instructions**	⓪ ① ② ③ ④ ⑤ ⑥ ⑦ ⑧ ⑨ ⑩
**11. If *antibiotics* are consumed in excess, they won't work when they are really needed**	⓪ ① ② ③ ④ ⑤ ⑥ ⑦ ⑧ ⑨ ⑩
**12. I prefer to keep *antibiotics* at home in case there is a need for them later**	⓪ ① ② ③ ④ ⑤ ⑥ ⑦ ⑧ ⑨ ⑩
**13. I trust the doctor's decision if s/he decides to prescribe or not prescribe *antibiotics***	⓪ ① ② ③ ④ ⑤ ⑥ ⑦ ⑧ ⑨ ⑩
**14. If I believe that I need an *antibiotic* and the doctor did not prescribe it, I will get it at the pharmacy without a prescription**	⓪ ① ② ③ ④ ⑤ ⑥ ⑦ ⑧ ⑨ ⑩
**15. Doctors often explain clearly to the patient the reasons for prescribing or not prescribing *antibiotics***	⓪ ① ② ③ ④ ⑤ ⑥ ⑦ ⑧ ⑨ ⑩
**16. Doctors often explain clearly to the patient the instructions for the use of *antibiotics***	⓪ ① ② ③ ④ ⑤ ⑥ ⑦ ⑧ ⑨ ⑩
**17. When you buy *antibiotics*, the pharmacist tells you about the importance of correct therapeutic compliance/adherence**	⓪ ① ② ③ ④ ⑤ ⑥ ⑦ ⑧ ⑨ ⑩

18. In the past month, did you take *antibiotics*? ○ Yes (please move to question 19) ○ No (please move to question 29)

Answer the following questions about your consumption of *ANTIBIOTCS* IN THE PAST MONTH

19. How long was the duration of your last treatment with *antibiotics*?

Number of Days ☐☐ Number of Months ☐☐

20. Who prescribed or recommended you the use of *antibiotics*? (You can choose more than one answer)

- ○ The doctor
- ○ Friends
- ○ The family
- ○ The pharmacist
- ○ I had them at home

21. The last time you had to take *antibiotics*, did you complete the course of treatment?

- ○ Yes
- ○ No

22. What did you do with the *antibiotics* that were left unused? (You can choose more than one answer)

- ○ I kept them to be used the next time I am sick
- ○ I disposed of them in the garbage or sewage system
- ○ I gave them to someone else
- ○ I didn´t have any remaining *antibiotics*

23. The last time you had to take *antibiotics*, did you forget to take any of the doses?

- ○ Never (please move to question 25)
- ○ Sometimes (please move to question 24)
- ○ Often (please move to question 24)

24. What did you do when you skipped a dose of your *antibiotics*?

- ○ I continued the following doses normally
- ○ I doubled/increased the following dose
- ○ I took it as soon as I remembered

25. The last time you had to take *antibiotics*, did you change the dose on your own (without medical advice)?

- ○ Never (please move to question 29)
- ○ Sometimes (please move to question 26)
- ○ Often (please move to question 26)

26. What did you do when you changed your dose of antibiotics on your own (without medical advice)?

- ○ I took more of it (please move to question 27 then to question 29)
- ○ I took less of it (please move to question 28)
- ○ Sometimes I took more, and sometimes I took less of it (please answer questions 27 and 28)

27. Why did you increase the dose of *antibiotics* on your own (without medical advice)? (You can choose more than one answer)

- ○ I forgot to take the previous dose
- ○ I felt very sick
- ○ I didn't feel a notable improvement
- ○ I felt better and wanted to improve even more

28. Why did you reduce the dose of *antibiotics* on your own (without medical advice)? (You can choose more than one answer)

- ○ I was worried about the medicine's side effects
- ○ I was tired, and I forgot
- ○ I felt that I was taking too many medicines
- ○ I was in a hurry and I forgot
- ○ I don't like to take medicines at night
- ○ I was feeling better

Code of the questionnaire: ____________________ **Date:** ________________

General Demographic Characteristics

29. Indicate your gender ◯ Male ◯ Female

30. What is your date of birth? (Please write the corresponding numbers in the spaces below)

☐☐ ☐☐ ☐☐☐☐
Day **Month** **Year**

31. What is your highest educational level?
- ◯ I did not go to school
- ◯ Elementary (grade 1 to grade 6)
- ◯ Intermediate (grade 7 to grade 9)
- ◯ Secondary (grade 10 to grade 12)
- ◯ University

32. Are you currently working? ◯ Yes ◯ No

33. What does your health insurance cover? (You can choose more than one answer)
- ◯ I do not have any health insurance
- ◯ The cost or a part of the cost of medical consultations
- ◯ The cost or a part of the cost of my medications

34. Please indicate your marital status:
- ◯ Married (please move to question 35)
- ◯ Divorced (please move to question 37)
- ◯ Widowed (please move to question 37)
- ◯ Separated from your spouse, but not divorced (please move to question 35)

35. What is the highest educational level of your spouse?
- ◯ He/She did not go to school
- ◯ Elementary (grade 1 to grade 6)
- ◯ Intermediate (grade 7 to grade 9)
- ◯ Secondary (grade 10 to grade 12)
- ◯ University

36. Is your spouse currently working? ◯ Yes ◯ No

37. How many members live in your house, including you?
◯ 2 ◯ 3 – 4 ◯ 5 – 6 ◯ More than 6

38. Are there any health facilities (such as dispensaries or hospitals) located near your area of residence?
◯ Yes ◯ No

39. What is the total monthly family income (the total income earned/received by all family members living in your house)?
- ◯ Less than US$500 per month
- ◯ Between US$500 and US$999 per month
- ◯ Between US$1000 and US$1499 per month
- ◯ Between US$1500 and US$1999 per month
- ◯ Between US$2000 and US$2500 per month
- ◯ More than US$2500 per month

40. Do you consult a doctor when you are sick?
◯ Never ◯ Rarely
◯ Sometimes ◯ Always (please move to question 42)

41. What is the reason for not always consulting a doctor? (You can choose more than one answer)
- ◯ No need for a doctor
- ◯ Fear of doctors
- ◯ Unavailability of sufficient money
- ◯ Insufficient time to visit the doctor
- ◯ Long wait before being examined by the doctor
- ◯ Unavailability of a clinic near my house

42. Have you ever received a medical prescription over the phone?
◯ Yes ◯ No

43. Do you drink alcohol?
- ◯ Yes (please move to question 44)
- ◯ No (you have completed the questionnaire. Thank you!)

44. How often do you drink alcohol?
- ◯ On special occasions
- ◯ Once a week
- ◯ Only on weekends
- ◯ 3 – 5 times a week
- ◯ Every day

Thank you very much for your time and collaboration!
